# Supplementary material for: Increase in global emissions of HFC-23 despite near-total expected reductions
Source: Nat Commun. 2020 Jan 21;11:397. doi: 10.1038/s41467-019-13899-4 (PMC6972758; doi:10.1038/s41467-019-13899-4)
Supplement: Supplementary file 1 — Supplementary Information [file 41467_2019_13899_MOESM1_ESM.pdf]

Supplementary information
Increase in global emissions of HFC-23 despite near-total expected emission reductions

Stanley et al.

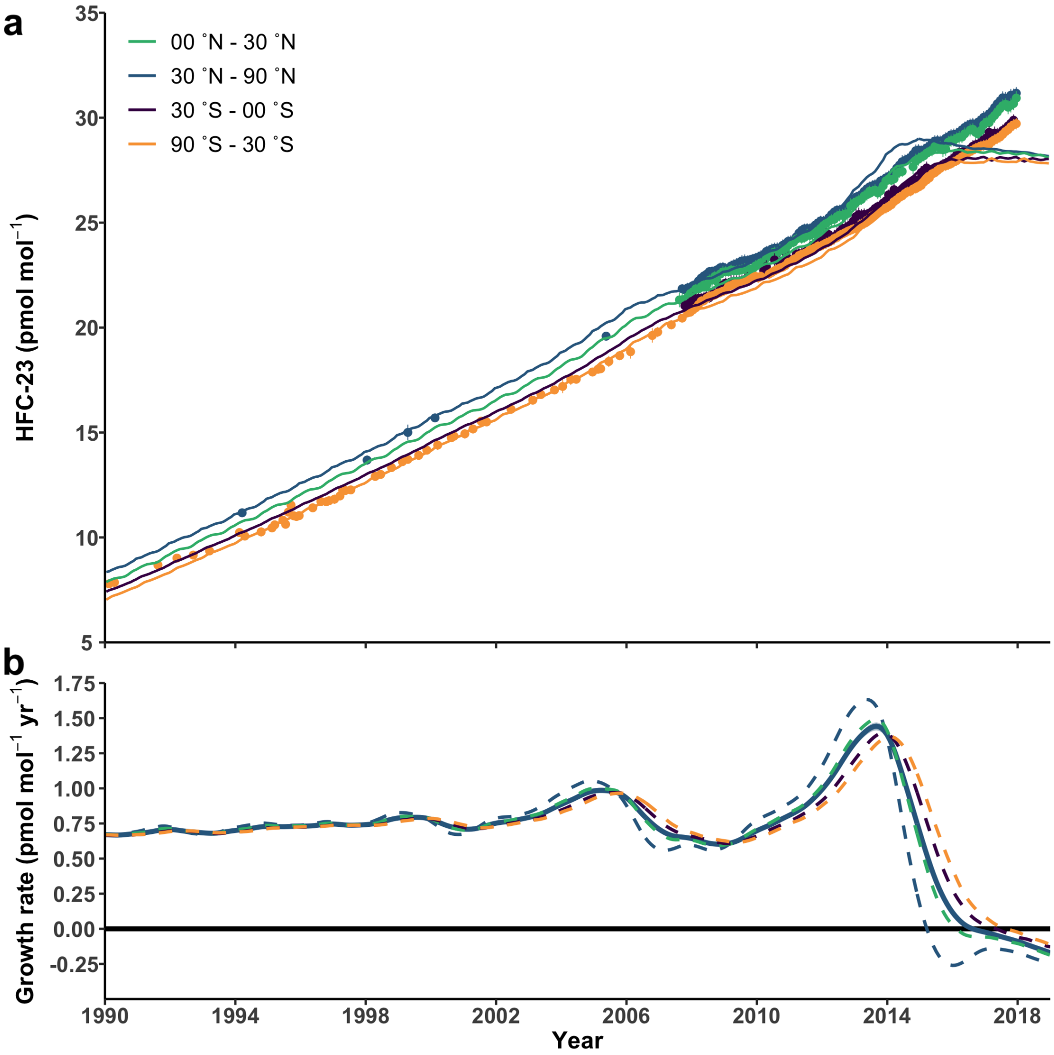

Supplementary Figure 1. HFC-23 observations, model-derived mole fractions and growth rates. a, Modelled HFC-23 mole fractions for the four equal-mass latitudinal subdivisions in the Advanced Global Atmospheric Gases Experiment 12-box model based on data from the Developing Country With Abatement global bottom-up emissions estimate (solid lines). In situ and archive air data are shown as dots (archive) or points with error bars (in situ, see Figure 2 in main text). An offset was applied to the model (primarily accounting for the influence of pre-1990 emissions) so that the 2014 annual mean emissions agree with the data. **b,** model-derived annual HFC-23 growth rates (global - blue solid line with 1-sigma uncertainty indicated by shading; dashed lines show semi-hemispheric growth rates) in pmol mol^{-1} yr^{-1}.

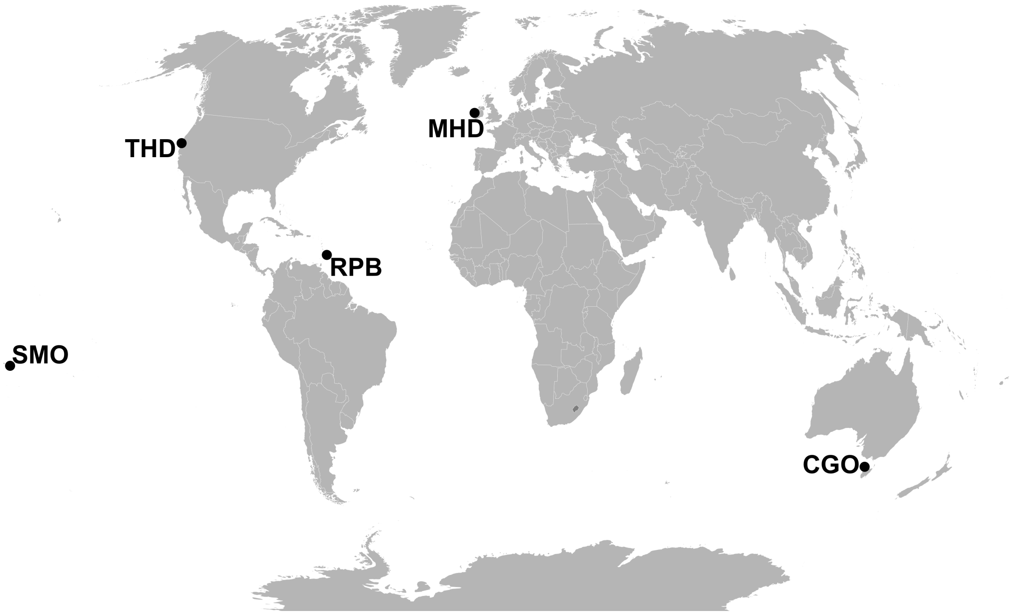

Supplementary Figure 2. Locations of the five measurement stations. The five core Advanced Global Atmospheric Gases Experiment measurement sites used in this study are located at Mace Head (MHD), Ireland; Trinidad Head (THD), California, USA; Ragged Point (RPB), Barbados; Cape Matatula (SMO), American Samoa and Cape Grim (CGO), Tasmania; Australia.

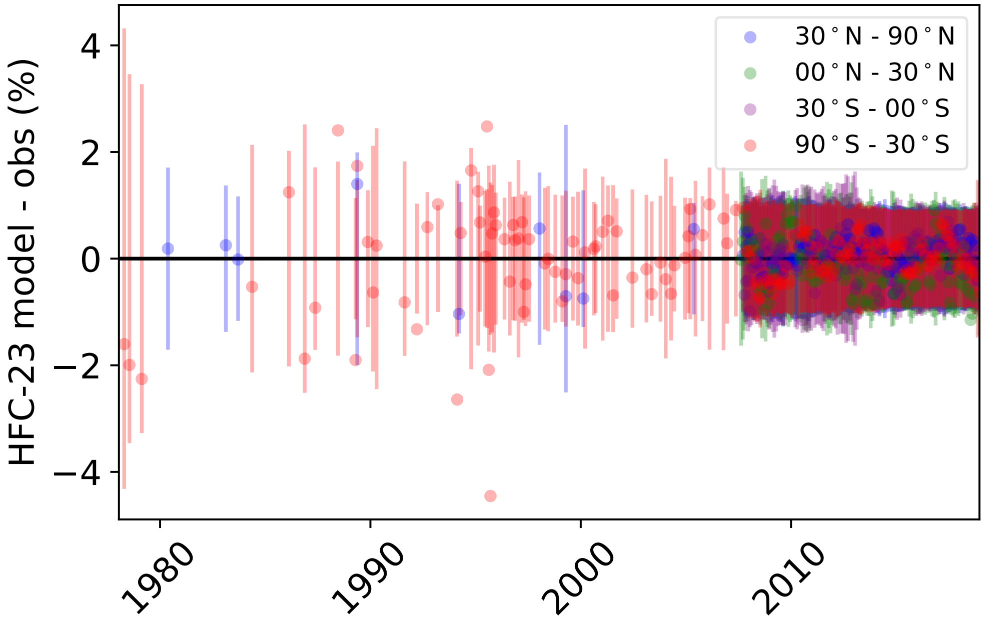

Supplementary Figure 3. HFC-23 model/measurement residual from the global inversion. Atmospheric data prior to 2007 uses measurements from the Cape Grim Air Archive and northern hemispheric historical flask samples; however, 2007 to present uses data from the 5 core Advance Global Atmospheric Gases Experiment background measurement stations.

Supplementary Table 1. **Bottom-up HFC-23 emissions to the atmosphere (Gg yr^{-1}).**

Year	Developing countries	Developed countries	Total	Developing countries + reported reductions	Total + reported reductions	2011 abatement maintained
1990	0.3	8.6	8.9	0.3	8.9	NA
1991	0.6	8.3	8.9	0.6	8.9	NA
1992	1.2	8.3	9.5	1.2	9.5	NA
1993	1.6	7.4	9.0	1.6	9.0	NA
1994	2.0	7.3	9.3	2.0	9.3	NA
1995	2.0	7.8	9.8	2.0	9.8	NA
1996	2.1	7.5	9.7	2.1	9.7	NA
1997	2.3	7.8	10.1	2.3	10.1	NA
1998	1.2	8.6	9.8	1.2	9.8	NA
1999	3.7	7.1	10.7	3.7	10.7	NA
2000	4.1	6.7	10.8	4.1	10.8	NA
2001	4.5	5.0	9.5	4.5	9.5	NA
2002	6.2	4.2	10.4	6.2	10.4	NA
2003	7.6	3.1	10.6	7.6	10.6	NA
2004	8.7	3.1	11.9	8.7	11.8	NA
2005	10.6	3.0	13.6	10.3	13.3	NA
2006	12.2	2.5	14.7	10.7	13.2	NA
2007	13.4	2.6	16.0	7.2	9.8	NA
2008	14.9	2.3	17.2	7.4	9.7	NA
2009	15.7	1.2	16.9	7.4	8.6	NA
2010	17.6	1.3	18.9	8.7	10.0	NA
2011	18.9	1.0	20.0	10.0	11.1	NA
2012	19.9	1.1	21.0	11.6	12.8	10.2
2013	18.8	1.2	20.0	16.1	17.3	10.8
2014	19.5	1.4	20.8	18.9	20.2	11.8
2015	15.6	1.1	16.6	9.4	10.5	7.6
2016	16.0	0.9	17.0	2.7	3.6	8.0
2017	17.8	1.7	19.5	0.7	2.4	10.6

Developed (Non-Article 5 under the Montreal Protocol; Annex I under the United Nations Framework Convention on Climate Change; UNFCCC; including Turkey but excluding Israel) countries data was compiled and aggregated from the 2019 National Inventory Reports submitted to the UNFCCC by each Annex I party[1].
Developing (Article-5 under the Montreal Protocol; non-Annex I under the UNFCCC; including Israel) countries' data are based on individual states HCFC-22 production multiplied by an emissions factor based on literature knowledge[2–5] and do not include any CDM abatement.
Total data is the summation of the developing and developed countries' data.
Developing countries + reported reductions and Total + reported reductions data include Clean Development Mechanism HFC-23 reductions (shown in Table 3) and reported reductions by China and India between 2015 and 2017 in the estimates[3, 4].
2011 abatement maintained outlines data where the maximum CDM reported reduction value (2011, shown in Table 3) is maintained and subtracted from the Total data (bottom up with no abatement).
NA's represent no data.

Supplementary Table 2. **Bottom-up total HCFC-22 production (Gg yr^{-1}) for developing and developed countries.**

Year	Developing countries	Developed countries	Total
1990	7.1	58.2	65.4
1991	17.5	131.4	148.9
1992	33.5	223.0	256.5
1993	43.4	282.6	326.0
1994	54.6	305.4	360.0
1995	54.6	356.9	411.5
1996	58.9	368.1	427.0
1997	62.9	369.2	432.1
1998	33.1	412.1	445.2
1999	100.7	394.3	495.0
2000	116.7	360.8	477.6
2001	126.9	352.6	479.5
2002	174.8	340.1	514.9
2003	214.5	332.9	547.3
2004	289.5	341.7	631.2
2005	350.9	349.4	700.3
2006	405.2	307.2	712.4
2007	471.3	327.0	798.3
2008	524.4	292.3	816.7
2009	558.0	195.8	753.8
2010	624.0	229.9	853.9
2011	671.5	241.8	913.3
2012	713.9	219.9	933.8
2013	675.3	193.5	868.9
2014	699.3	210.0	909.3
2015	603.8	225.2	829.0
2016	643.5	208.8	852.3
2017	725.3	221.8	947.1

Data was obtained from UNEP HCFC database[6] and comprises of aggregated total production (for feedstock and dispersive uses) data for developing (Article 5) and developed (non-Article 5) countries, as well as a global total.

Supplementary Table 3. **Country specific and total HFC-23 abatement (Gg yr^{-1}) during the CDM period.**

Year	China	India	Mexico	South Korea	Argentina	Total
2003	0.00	0.000	0.00	0.036	0.000	0.036
2004	0.00	0.019	0.00	0.036	0.000	0.054
2005	0.00	0.201	0.00	0.114	0.000	0.315
2006	0.31	0.851	0.11	0.186	0.000	1.453
2007	4.64	1.143	0.17	0.196	0.018	6.171
2008	5.65	1.373	0.22	0.193	0.089	7.522
2009	6.45	1.412	0.20	0.195	0.128	8.384
2010	6.92	1.376	0.23	0.207	0.157	8.887
2011	6.94	1.487	0.23	0.193	0.149	8.995
2012	6.44	1.399	0.18	0.103	0.152	8.274
2013	2.48	0.132	0.00	0.000	0.033	2.641
2014	0.61	0.000	0.00	0.000	0.000	0.607

Manufacturer specific data are compiled from 474 Clean Development Mechanism (CDM) monitoring reports and are available at https://cdm.unfccc.int/Projects/registered.html.
Total annual mass destroyed of HFC-23 for each facility was compiled from the CDM reports and then aggregated for each country.
Total CDM abatement is the summation of all of the listed country data.

Supplementary Table 4. Top-down HFC-23 emissions to the atmosphere (Gg yr^{-1}) from atmospheric observations and the 2-D AGAGE 12-box model.

Year	Global annual emissions	16th Percentile	84th Percentile
1990	7.0	6.4	7.7
1991	7.0	6.3	7.6
1992	7.4	6.8	8.1
1993	7.9	7.3	8.6
1994	8.3	7.7	8.9
1995	8.9	8.3	9.6
1996	9.6	9.0	10.3
1997	10.1	9.5	10.7
1998	10.3	9.6	11.1
1999	10.8	10.1	11.6
2000	10.3	9.5	11.1
2001	9.3	8.6	10.1
2002	9.5	8.7	10.2
2003	10.3	9.5	11.1
2004	11.8	11.0	12.6
2005	13.2	12.4	14.0
2006	13.3	12.6	14.1
2007	11.7	11.0	12.4
2008	11.3	10.7	11.9
2009	9.6	9.1	10.2
2010	10.4	9.9	11.0
2011	11.6	11.0	12.3
2012	12.9	12.3	13.5
2013	14.0	13.3	14.5
2014	14.5	13.8	15.1
2015	13.1	12.5	13.8
2016	12.9	12.3	13.6
2017	14.9	14.2	15.6
2018	15.9	14.9	16.7

* Note: Data are tabulated as annual mean mid-year values.

Supplementary References

1. United Nations Framework Convention on Climate Change. *National Inventory Submissions 2019.* https://unfccc.int/process-and-meetings/transparency-and-reporting/reporting-and-review-under-the-convention/greenhouse-gas-inventories-annex-i-parties/national-inventory-submissions-2019. (2019).
2. Simmonds, P. G. *et al.* Recent increases in the atmospheric growth rate and emissions of HFC-23 (CHF_3) and the link to HCFC-22 ($CHClF_2$) production. *Atmos. chem. Phys.* **18,** 4153–4169 (2018).
3. United Nations Environment Programme. *Key aspects related to HFC-23 by-product control technologies.* (United Nations Environment Programme, Montreal, 2017).
4. United Nations Environment Programme. *Cost-effective options for controlling HFC-23 by-product emissions.* (United Nations Environment Programme, Montreal, 2018).
5. United Nations Environment Programme. *Corrigendum: Cost-effective options for controlling HFC-23 by-product emissions.* (United Nations Environment Programme, Montreal, 2018).
6. United Nations Environment Programme. *Countries.* https://ozone.unep.org/countries. (2019).
